# PockFlex: a web server for flexibility-aware binding site identification and prioritisation from structural ensembles

**DOI:** 10.1093/nar/gkag453

**Published:** 2026-05-08

**Authors:** Inés S Rahali, Yacine Serir, Kheira Rahali, Delphine Flatters, Leslie Regad, Anne-Claude Camproux

**Affiliations:** Inserm U1133, CNRS UMR 8251, Université Paris Cité, Rue Hélène Brion, 75013 Paris, France; IT Consulting Department, Data Gorillas, Place du President Thomas Woodrow Wilson, 31000 Toulouse, France; IT Consulting Department, Data Gorillas, Place du President Thomas Woodrow Wilson, 31000 Toulouse, France; Inserm U1133, CNRS UMR 8251, Université Paris Cité, Rue Hélène Brion, 75013 Paris, France; Inserm U1133, CNRS UMR 8251, Université Paris Cité, Rue Hélène Brion, 75013 Paris, France; Inserm U1133, CNRS UMR 8251, Université Paris Cité, Rue Hélène Brion, 75013 Paris, France

## Abstract

PockFlex is a web server designed to analyse pockets across protein structural ensembles and support the reconstruction, characterisation, and prioritisation of recurrent binding site organisations. Applicable to ensembles derived from molecular dynamics simulations, multiple experimental structures, or protein structure predictions, PockFlex detects pockets independently in each conformation, retains those overlapping a user-defined region of interest, and groups them across the ensemble by residue-level similarity. This residue-centred clustering framework identifies recurrent binding site clusters, quantifies residue recurrence and variability, and distinguishes persistent from transient binding site regions across the ensemble. Pocket-level druggability, predicted using the PockDrug workflow, is summarised at the cluster level to support binding site prioritisation under conformational variability while preserving access to individual pocket scores. The web application provides interactive, residue-level insights into pocket organisation, variability, and druggability in structural ensembles. The web server is free and open to all users, without login requirement, at https://pockflex.rpbs.univ-paris-diderot.fr/.

## Graphical Abstract

**Graphical Abstract:**
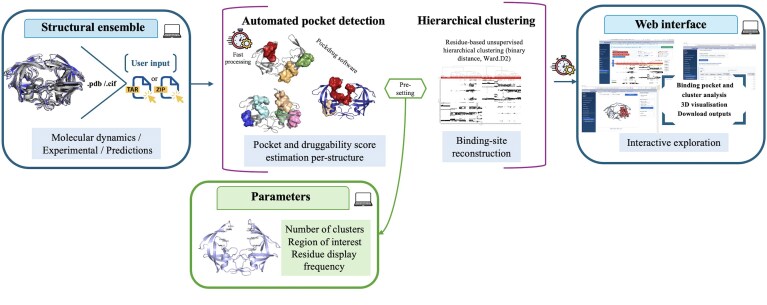

## Introduction

The identification of protein binding sites is a fundamental step in structure-based drug design. Binding pockets are commonly identified as cavities on protein surfaces that can accommodate small molecules, often using geometry-based methods applied to individual protein conformations. Numerous tools have been developed for pocket detection on individual structures [1, 2], including geometry-based and machine-learning approaches such as Fpocket [3] and P2Rank [4]. In parallel with pocket detection, the prioritisation of candidate binding regions increasingly relies on druggability assessment [5–7].

However, proteins are inherently dynamic, and functionally relevant binding regions may undergo conformational changes that alter pocket shape, accessibility, continuity, and druggability. This motivates methods able to analyse pocket organisation across structural ensembles rather than within a single conformation [8]. It has recently been shown that protein conformational diversity can substantially influence druggability assessments beyond single-structure analyses [9]. More broadly, recent in silico approaches combine geometry-based pocket detection with sequence-based and structure-based binding site prediction, as well as machine-learning and deep-learning methods [8]. Recent developments such as AlphaFold 3 have further expanded the landscape of structure prediction and binding site inference [10].

Fewer methods have been developed to analyse pockets across conformational ensembles. In particular, MDpocket [11], TRAPP [12], and D3Pockets [13] provide valuable information on pocket dynamics, persistence, continuity, and transient subpocket behaviour across multiple conformations. By contrast to volumetric or continuity-based analyses, PockFlex relies on pocket detection performed independently in each conformation, followed by residue-based clustering to reconstruct recurrent binding site organisation (see Supporting material S1, Supplementary Table S1).

PockFlex flexibility-aware web server is designed to support the reconstruction, characterisation, and prioritisation of recurrent binding site clusters from structural ensembles. It processes ensembles derived from molecular dynamics trajectories, multiple experimental structures, or predicted conformations, provided that residue numbering remains consistent across the ensemble. Each conformation is processed independently with the published PockDrug framework [14], which relies on an Fpocket-based geometric procedure for pocket estimation and assigns pocket-level druggability scores. Pockets are then filtered according to their overlap with a user-defined region of interest (ROI), i.e., a set of residues delimiting the structural region to be explored, and grouped across conformations into recurrent binding site clusters based on residue-level similarity. This enables multiple pocket configurations to be analysed and interpreted across the ensemble.

Rather than introducing a new druggability predictor, PockFlex leverages PockDrug-based pocket-level estimates across conformational ensembles to support the interpretation and prioritisation of recurrent clusters. Cluster-level druggability is interpreted as a summary of the prevalence of favourable conformations, while pocket-level scores and categories remain available for user-guided interpretation.

In this framework, the ROI should not be interpreted as a predefined binding site, but as a user-defined exploration scope. This design enables first-pass exploration of broad functional regions followed by iterative refinement of more focused regions when recurrent or promising clusters are identified. The methodology later formalised in PockFlex builds on previously published applications to biologically relevant systems, including the influenza A virus NS1 RNA-binding domain and the SARS-CoV-2 spike receptor-binding domain [15, 16]. By explicitly integrating structural variability into binding site identification, characterisation, and prioritisation, PockFlex enables the analysis of persistent, transient, and rare sites that may remain inaccessible from individual static structures, together with their associated residue variability. The web application provides interactive visualisation tools, residue-level stability maps, and cluster-level druggability summaries to support binding site prioritisation in early-stage drug discovery.

## Materials and methods

Each PockFlex job follows a two-step workflow: per-conformation pocket analysis followed by ensemble-level pocket clustering for binding site identification.

### Pocket analysis at the conformation level

For each structure in the ensemble, PockFlex performs four successive steps.

1. **Pocket estimation**. Candidate pockets are estimated independently for each conformation using the published PockDrug workflow, which relies on an Fpocket-based geometric procedure [3]. This ligand-independent strategy is applicable to both apo and holo protein structures.
2. **Pocket characterisation**. For each detected pocket, geometric, physicochemical, and residue-composition descriptors are computed. Pocket composition is represented as the list of pocket-lining residues identified by amino-acid type and residue number, together with conformation and pocket-level identifiers enabling pocket tracking across the ensemble.
3. **Pocket-level druggability estimation**. PockFlex relies on the published PockDrug/PockDrug-Server framework for pocket-level druggability estimation [6, 14]. For each detected pocket, PockDrug returns a continuous druggability score, d; when a binary interpretation is required, pockets with *score_d*$\ge$ 0.50 are considered druggable. In PockFlex, these pocket-level predictions are preserved and summarised at the cluster level as the proportion of pockets predicted as druggable within a given binding site cluster. Cluster-level druggability should therefore be interpreted as a summary of favourable conformations rather than as a replacement for pocket-level scores, while the four-level PockDrug categorisation remains available.
4. **Pocket filtering**. Only pockets involving at least eight pocket-lining residues are retained for downstream analysis. Among these, PockFlex retains all pockets including at least one residue of the user-defined ROI, that is, a set of residues focusing on a minimal structural region to be explored. The retained ROI-overlapping pockets are then pooled across the ensemble for residue-based comparison and binding site cluster reconstruction.

### Binding site assembly: pocket clustering

All retained pockets are compared to identify recurrent binding site organisation across structural ensembles. In an earlier approach [17], pocket similarity was quantified using Euclidean distance computed on global PockDrug descriptors, which proved insufficient to track conserved pockets or sub-pockets along conformational dynamics. We therefore adopted a residue-centered pocket comparison strategy, in which pocket similarity is quantified using binary residue fingerprints and the asymmetric binary distance implemented in R. This dissimilarity is bounded between 0 and 1 and evaluates residue mismatches while disregarding shared absences, which is appropriate for residue presence/absence data.

This residue-based distance is not intended as a standalone predictor of binding site identity or biological distinctness, but as a practical similarity measure for grouping compositionally related pockets. Hierarchical clustering is then performed using Ward.D2 [18, 19] as the default agglomeration rule, used here as a practical criterion to obtain compact and interpretable groups within a multi-resolution framework.

The resulting dendrogram provides a structured representation of residue-level relationships among pockets across the ensemble: closely related pockets merge at low dendrogram heights, whereas more heterogeneous pockets are separated at higher levels. A first run is performed to estimate ROI-overlapping pockets across the ensemble. A heuristic clustering value, K, is then proposed to the user as the average number of ROI-overlapping pockets detected per conformation, although users may also define K directly. This value should be interpreted as a starting resolution parameter rather than as an optimal estimate of the true number of binding site clusters. The resulting clustering groups pockets with similar residue composition and thereby identifies recurrent binding site organisations across the conformational ensemble.

The value of K controls clustering granularity and can be adjusted either to resolve sub-clusters within flexible binding sites or to merge closely related binding regions. Dendrograms combined with residue-based heatmaps support the assessment of cluster coherence by revealing similarities and differences in residue composition across pockets. Accordingly, cluster interpretation in PockFlex does not rely on the distance metric alone, but on its combination with cluster recurrence across the ensemble, residue-frequency structure within clusters, and multi-resolution exploration. Dedicated sensitivity and size-bias controls supporting this clustering formulation and robustness against structural noise and pocket-detection variability were further assessed by repeated conformational subsampling, as detailed in Supporting material S2, Supplementary Tables 2–S5, Supplementary Figs S1–S2.

### PockFlex application implementation

PockFlex is implemented using the Django web framework [20], with a PostgreSQL database [21] for the backend. The user interface relies on custom TailwindCSS [22], standard JavaScript, integrates 3Dmol.js [23] for interactive three-dimensional visualisation. Backend processing integrates established computational tools within a reproducible R-based workflow. All scripts are containerised using Docker [24] to ensure dependency coherence, portability, and reproducibility of the server-side workflow. Local deployment is currently not supported.

Each conformation is processed independently with the published PockDrug workflow, which estimates candidate pockets through an Fpocket-based geometric procedure and assigns pocket-level descriptors and druggability scores. ROI-overlapping pockets are then selected and clustered across the ensemble using custom R scripts. Intermediate results are stored in RData format for efficient downstream processing.

Input files are managed through Django’s file handling system, with support for Protein Data Bank (PDB) [25] and Crystallographic Information File (CIF) [26] formats. Compressed files are automatically decompressed, and CIF files are converted to PDB format using Biopython [27] before analysis. Session identifiers generated with the Sqids library allow users to retrieve analyses through unique session hashes. Job monitoring is performed by regular polling of the processing pipeline, with real-time updates communicated to the interface. PockFlex is freely accessible without registration on the RPBS platform hosted at Université Paris Cité (https://bfa.u-pariscite.fr/en/rpbs-platform/).

PockFlex currently accepts up to 500 conformations per job, with up to 300 residues per ROI and a maximum compressed upload size of 100 MB. Typical processing times depend on ensemble size: pocket detection averages 1.2 s per structure, and clustering usually completes within 1–2 min for standard jobs on the current institutional deployment. Jobs are processed through a queued backend with adaptive parallelisation (3, 6, or 8 workers depending on job size and server load). Anonymous sessions are retained for 72h.

### The PockFlex web application interface

**Figure 1.**
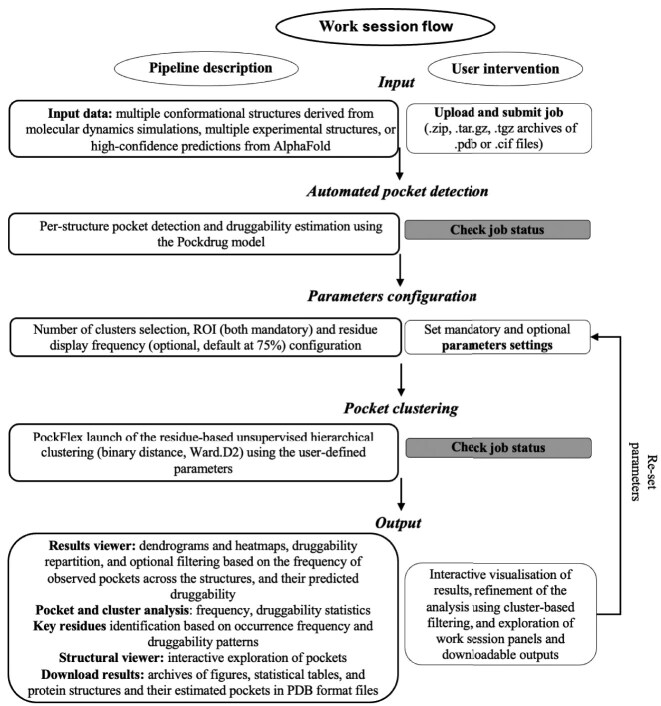
PockFlex analysis pipeline for ensemble-based pocket detection and clustering. The workflow highlights automated processing steps and user interventions from data upload to interactive result exploration.

PockFlex provides an interactive, session-based web interface for the exploration, analysis, and prioritisation of binding sites across protein structural ensembles. As summarised in Fig. 1, the interface guides the user from data upload and parameter definition to clustering, visualisation, and result download.

#### Work sessions and inputs

Each analysis is performed within a temporary anonymous work session identified by a unique session ID, which enables users to retrieve their results during the retention period. Users upload a single compressed archive containing protein conformations in PDB or CIF format. Input files are automatically validated for format and content consistency. PockFlex assumes an input ensemble prepared so that residue correspondence remains meaningful across conformations. The server does not reconstruct missing segments, resolve alternate conformations, or correct incomplete side-chain modelling; such preprocessing steps must therefore be handled upstream. All conformations must share a consistent residue numbering scheme to enable pocket comparison across the ensemble. Users then define the analysis parameters, including the ROI, the clustering resolution, and an optional residue display frequency threshold.

#### Job execution and monitoring

After upload, pocket estimation is first performed independently for each conformation. Once this step is completed, the interface prompts the user to set clustering parameters before launching the ensemble-level clustering workflow. Job progression is updated in real time through the interface until completion or failure.

#### Outputs and interactive exploration

Results are displayed through multiple coordinated panels, including global pocket and druggability summaries, cluster-level statistics, residue-frequency analyses, and interactive structural visualisation. Hierarchical clustering results are shown as dendrograms and heatmaps, and cluster-level druggability is summarised from pocket-level predictions. Users can further explore the results through frequency and druggability-based filtering, inspect key residues within clusters, and visualise pockets on the structures. All clusters are displayed by default, and filtering by cluster-level druggability is left to the user rather than applied as an automatic exclusion rule, so clusters containing only a minority of highly druggable conformations remain accessible for inspection. Results are returned through the interactive interface together with downloadable CSV tables, graphical outputs, and PDB files; no separate user-facing log file is currently generated.

#### Practical interpretation and prioritisation of binding sites

PockFlex does not require a predefined binding site as input. Instead, users define a ROI as a set of residues delimiting the structural region to explore; the ROI should therefore be understood as an exploration scope rather than as a predefined pocket or binding site. Pockets are detected independently in each conformation and those including at least one residue from the ROI are clustered according to residue-level similarity. This clustering enables the identification, interpretation, and ranking of recurrent binding site clusters across the structural ensemble. Binding site prioritisation is primarily guided by the combination of cluster recurrence and predicted druggability, complemented by residue-level frequency analyses and interactive three-dimensional visualisation. Practical guidance for ROI definition, parameter interpretation, and iterative analysis is provided in Supporting material S3 and Supplementary Table S6.

In practice, clusters exhibiting high recurrence and a high prevalence of favourable conformations across the ensemble represent robust candidates for downstream analysis. PockFlex can also be used iteratively, by first defining a broad ROI, then exploring different K values, and finally refining promising regions for more focused analysis. Conversely, low-frequency clusters should be interpreted more cautiously, particularly when derived from limited sampling or insufficient ensemble diversity. Cluster-level druggability should be interpreted as a summary of the prevalence of favourable conformations within a cluster, whereas the underlying pocket-level scores remain available to assess intra-cluster heterogeneity and to identify rarer but potentially highly druggable states.

Although pockets are initially detected at the atomic level, clustering in PockFlex is performed at the residue level. As a result, pockets with slightly different atomic definitions may still be grouped together if they share a similar residue composition.

### Application example

PockFlex has been used to characterise the pocket landscape of HIV-1 protease (PR1), a well-established drug target whose activity depends on large-amplitude flap motions controlling access to the active site [28–30].

**Figure 2.**
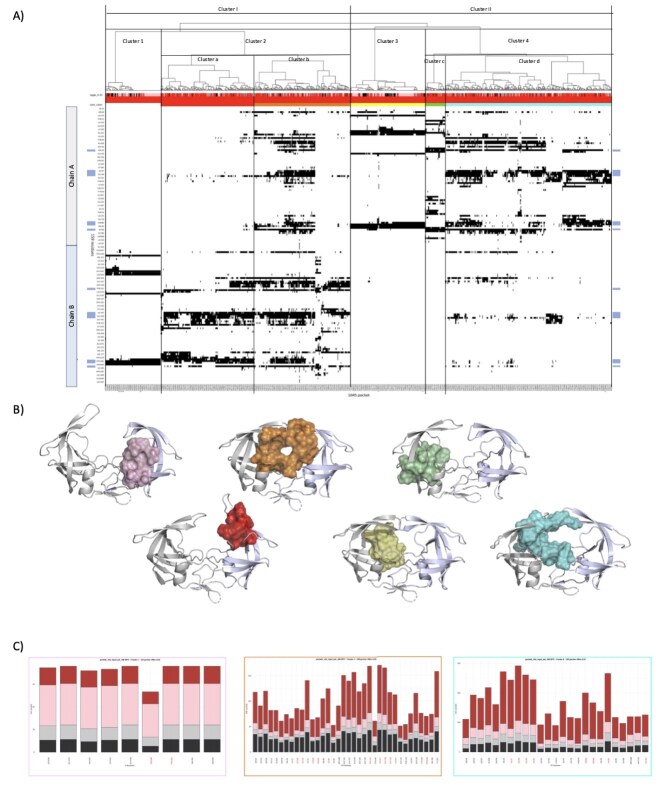
Multi-resolution PockFlex analysis of ROI-overlapping pockets in HIV-1 protease PR1. (**A**) Hierarchical clustering of pockets shown as a dendrogram with a residue–pocket presence heatmap. The top annotation bar reports pocket-level PockDrug druggability for each pocket conformation, and the second bar indicates cluster assignment. (**B**) Representative structural localisations of selected clusters on the PR1 dimer. (**C**) Cluster-level residue-frequency profiles showing residue conservation together with the distribution of PockDrug druggability categories among pocket conformations assigned to clusters 1, 2b, and 4d.

To illustrate the intended exploratory workflow, the analysis was first performed using a broad ROI encompassing the entire protein, across 501 frames extracted from a 500 ns molecular dynamics simulation. PR1 structure preparation, together with the different ROI definitions and corresponding cluster analyses, are provided in Supporting material S4. In total, 2695 pockets were identified and grouped according to residue-composition similarity, revealing a structured and heterogeneous pocket landscape (see global clustering in Supplementary Fig. S3). The central binding site, identified by the presence of catalytic residues at the dimer interface, was clearly recovered without prior restriction to a local binding region. However, several clusters involving catalytic residues displayed distinct surrounding residue compositions, consistent with multiple conformational states of the central pocket associated with flap dynamics, whereas a smaller number of clusters involved conserved regions such as the fulcrum and cantilever, highlighting alternative pocket organisations independent of the active site.

A second analysis, using a focused ROI including 16 residues from the central binding region (32, 47–50, 80, 81, 84, 132, 147–150, 180, 181, 184), identified 1045 ROI-overlapping pockets covering 91% of the trajectory. At low resolution, the classification separated pockets according to their dominant chain contribution (Fig. 2A). Partitioning into four clusters showed that each main group was further subdivided into a highly populated central-site cluster and a less populated lateral cluster in the fulcrum and wall regions. These lateral pockets were located on the outer surface of the protein (Fig. 2B), observed in both chains A and B, and recurred in 23% and 31% of frames, indicating that they are not rare events. Their conserved residue composition suggests limited structural variability (Fig. 2C), while their location near the fulcrum points to possible allosteric relevance.

Refining the classification into six clusters further resolved the conformational diversity of the central binding site (Fig. 2A). Two clusters corresponded to central pockets at the dimer interface: a canonical symmetric state involving residues from both chains (36% of frames) and a more populated asymmetric state dominated by one chain (57%), consistent with asymmetric flap motions. Their structural localisations are illustrated in Fig. 2B, whereas Fig. 2C highlights the greater residue conservation of the symmetric state and the higher variability of the asymmetric state. Consistent with previous D3Pockets analyses [13], the central binding site was recovered here as a stable region modulated by flap motions. However, whereas D3Pockets primarily emphasises pocket continuity, persistence, and geometric evolution along MD trajectories, PockFlex provides a complementary ensemble-level interpretation centred on recurrent residue-defined pocket states and their relative prevalence across the conformational ensemble. Additional chain-specific clusters included a recurrent lateral pocket at the flap–wall interface, consistent with the previously described eye pocket, and a less frequent pocket in the fulcrum/cantilever region compatible with a transient or cryptic cavity. Overall, this analysis shows that PR1, with a deprotonated catalytic Asp25 in chain B, explores a diverse ensemble of pocket conformations, including flexible central-site states, recurrent lateral pockets, and less populated chain-specific cavities.

## Discussion

The hierarchical clustering implemented in PockFlex provides a dichotomous organisation of pockets based on increasing residue-level dissimilarity, yielding a dendrogram that captures the diversification of pocket conformations across structural ensembles. On this basis, PockFlex is designed to support the exploration and prioritisation of recurrent, residue-defined binding site organisations under conformational variability, rather than simply identifying isolated pockets in static structures. By enabling ensemble-level analysis, the server provides a framework in which pocket recurrence, residue coherence, structural variability, and druggability can be considered jointly.

A key interest of this framework is its exploratory use. PockFlex does not require a predefined binding site, but allows users to start from a broad region of interest and progressively refine the analysis toward more focused regions and alternative clustering resolutions. This iterative strategy can help recover known binding regions while also examining neighbouring, partially overlapping, or less populated pocket states. In this context, highly recurrent clusters associated with conserved residue compositions and a high prevalence of favourable conformations may represent plausible candidates for downstream analysis, whereas less recurrent clusters should be interpreted more cautiously. Residue-frequency analyses further help distinguish more stable contributors from more variable or peripheral ones within each cluster. They may also help relate changes in residue composition across clusters to differences in predicted druggability profiles.

Compared with approaches that primarily analyse pocket continuity, persistence, or geometric evolution across conform-ational ensembles, PockFlex provides a complementary perspective centered on recurrent pocket states defined at the residue level. Since pockets are first detected independently in each conformation and then clustered at the residue level, pockets involving similar sets of residues can be grouped together even if their exact shape or position varies locally. PockFlex therefore complements, rather than replaces, volumetric or continuity-based approaches by offering an ensemble-level interpretation of recurrent binding site organisations and their relative prevalence across conformations.

More generally, the interpretation of PockFlex results depends on the quality, diversity, and representativeness of the input conformational ensemble, as well as on the consistency of structural data preparation, including residue numbering, chain annotation, structural completeness, and related preprocessing choices. In addition, clustering depends directly on upstream pocket detection, so changes in pocket-detection settings may affect the number, composition, and recurrence of the resulting clusters. Likewise, the druggability information provided by PockFlex should be interpreted as a guiding predictive layer rather than as experimental evidence: pocket-level scores and cluster-level summaries are intended to assist prioritisation and comparative interpretation across conformational states, but do not by themselves establish ligandability or functional relevance. The current validation of PockFlex is based on published application studies and supplementary robustness analyses. These elements support the practical relevance and interpretability of the workflow, although they do not yet constitute a broad benchmark across diverse annotated protein families or community-standard datasets, nor a head-to-head quantitative comparison with all established ensemble-aware pocket-analysis methods.

Within this scope, the present examples and literature-supported case studies support the practical relevance and interpretability of the workflow, including consistency with previously described functional or binding regions in distinct systems. Future developments may include comparative analyses across distinct conformational ensembles, sequence variants, or mutant structures, as well as the use of residue-frequency patterns to distinguish more conserved cluster cores from more variable peripheral regions.

## Supplementary Material

gkag453_Supplemental_File

## Data Availability

The data underlying this article are available and can be downloaded as a .zip archive directly from the web interface at https://pockflex.rpbs.univ-paris-diderot.fr/work-sessions.html.
