# Supplementary material for: PockFlex: a web server for flexibility-aware binding site identification and prioritisation from structural ensembles
Source: Nucleic Acids Res. 2026 May 8;54(W1):W295–300. doi: 10.1093/nar/gkag453 (PMC13355080; doi:10.1093/nar/gkag453)
Supplement: gkag453_Supplemental_File [file gkag453_supplemental_file.pdf]

Supporting material S1. Comparative positioning of PockFlex relative to representative ensemble-aware tools

Scope and rationale of the comparison

This supporting material is intended to clarify the methodological positioning of PockFlex with respect to a few recent representative ensemble-aware pocket analysis tools that account for structural flexibility across conformational ensembles, including molecular dynamics trajectories. It should not be interpreted as a formal benchmark of relative performance or predictive superiority, since the compared methods do not fully address the same analytical objective and do not generate directly equivalent outputs.

More specifically, PockFlex is positioned as a residue-centred ensemble-level workflow for reconstructing and prioritising recurrent binding-site clusters from independently detected pocket instances across conformational ensembles. By contrast, representative ensemble-aware tools such as MDpocket, TRAPP, and D3Pockets mainly emphasise pocket dynamics, persistence, continuity, or transient subpocket behaviour. The purpose of the present comparison is therefore to clarify methodological scope and complementarity, rather than to rank methods or to imply that they solve exactly the same task.

To clarify the scope of the contribution, we positioned PockFlex relative to representative ensemble-aware pocket analysis tools according to their primary analytical emphasis in Table S1.

Method	Main analytical object	Main representation across conformations	Primary analytical emphasis
**MDpocket**	Binding-site and channel tracking across conformational ensembles	Grid-based pocket density map built from α-sphere accumulation	Pocket occupancy, persistence, & cavity evolution
**TRAPP**	Analysis of transient binding pockets and subpockets, including conserved/transient pocket regions & contributing residues	Grid-based pocket shape and physicochemical descriptors with conserved vs transient regions/subpockets	Pocket modulation, flexibility, transient regions, subpocket opening, & variation
**D3Pockets**	Systematic analysis of dynamic pocket properties	Grid-point frequency and dynamic pocket-property profiles across snapshots	Pocket splitting, merging, continuity & transient subpocket
**PockFlex**	Recurrent residue-defined binding-site cluster reconstruction & prioritisation	Residue-level clustering of independently detected pockets; dendrogram and cluster summaries	Ensemble-level reconstruction, characterization & prioritization of recurrent binding site cluster

Table S1. Comparative methodological positioning of PockFlex relative to representative ensemble-aware pocket analysis tools. This table summarises the comparative methodological positioning of PockFlex relative to representative ensemble-aware tools. It is intended to clarify analytical scope and complementarity, not to provide a formal benchmark of relative performance. References for the representative ensemble-aware tools discussed in this table are provided in the main text and are repeated here for convenience: MDpocket [1], TRAPP [2], and D3Pockets [3].

Supporting material S2. Methodological validation of PockFlex clustering on HIV-1 protease (PR1)

S2.1. Scope and rationale

This supporting section examines the methodological robustness of the PockFlex clustering strategy on the PR1 benchmark case. The analyses are organised into two complementary parts. S2.2 addresses clustering resolution, that is, how the organisation of the PR1 pocket space changes across dendrogram cuts. S2.3 then examines the clustering formulation itself, including the influence of distance and linkage choices, as well as possible biases related to pocket size or residue count. Unless otherwise stated, all analyses were performed from the same PR1 pocket-by-residue binary matrix used for the reference clustering. The reference setting corresponds to the binary + Ward.D2 formulation, and comparisons were made either across clustering resolutions or against methodological variants relative to this same framework. Overall, these analyses address three practical questions: whether the recovered pocket organisation is stable across resolutions, whether the main clusters remain detectable under moderate perturbations of the structural ensemble, and whether the observed clustering reflects residue composition rather than trivial size effects.

All analyses were performed from the same PR1 pocket-by-residue binary matrix used for the reference clustering. Pairwise pocket dissimilarities were computed in R using stats::dist(method = "binary"), corresponding to the asymmetric binary distance for presence/absence data, commonly interpreted as a Jaccard-type dissimilarity because shared absences do not contribute to similarity. The reference hierarchical clustering was then performed with stats::hclust(method = "ward.D2"). Concordance between partitions was quantified using the Adjusted Rand Index (ARI) and the Normalized Mutual Information (NMI).

S2.2. Clustering resolution on the PR1 benchmark case

S2.2.1. Sensitivity of clustering resolution to the dendrogram cut

To assess how clustering resolution affects the organisation of the PR1 pocket space, we examined successive partitions obtained by cutting the same reference dendrogram from K = 2 to K = 6. Across this range, increasing K did not produce an abrupt reorganisation of pocket space, but rather a progressive hierarchical refinement of the same global organisation. The lowest-resolution partition (K = 2) was retained as the default coarse-grained view because it already captures the dominant asymmetry of the dimer, which represents the first large-scale organisational signal of the pocket landscape. Increasing K then progressively subdivided the major pocket clusters into finer subclusters while preserving this global structure. Within this progression, K = 4 was retained as a practically useful intermediate level for interpretation, because it already yields four well-individualised and substantial groups while remaining easy to interpret. However, this choice should not be understood as defining a uniquely optimal partition, and K = 5 can also be viewed as a closely related but slightly finer subdivision of the same global organisation. Higher values, including K = 6, mainly refine pre-existing clusters

rather than defining a different organisation of pocket space. Practically, this analysis shows that changing K does not arbitrarily reshape the PR1 pocket landscape. Instead, K mainly controls the level of resolution at which a stable underlying organisation is viewed. The corresponding cluster sizes, main subdivision events, and concordance values across successive cuts are summarised in Table S2. Taken together, these results indicate that the dendrogram cut primarily controls the level of structural resolution rather than the underlying organisation itself, supporting the interpretation of K as a parameter for multi-scale exploration rather than as a uniquely optimal clustering threshold. Accordingly, K = 2 is retained as the default coarse-grained entry point, K = 4 as the main intermediate resolution for practical interpretation, and K = 6 as an additional illustrative level for visualising finer hierarchical subdivision.

Resolution	Cluster sizes	Main split at next level	ARI	NMI
K = 2	484, 457	457 → 313 + 144	0.796	0.821
K = 3	484, 313, 144	484 → 375 + 109	0.799	0.879
K = 4	375, 313, 144, 109	313 → 274 + 39	0.942	0.953
K = 5	375, 274, 144, 109, 39	375 → 190 + 185	0.785	0.910
K = 6	274, 190, 185, 144, 109, 39	–	–	–

Table S2. Sensitivity of PR1 clustering to the dendrogram cut (K = 2 to K = 6). Cluster sizes are reported for successive partitions obtained by cutting the same reference dendrogram from K = 2 to K = 6 for the PR1 illustration dataset used in the manuscript. The "Main split at next level" column indicates the principal subdivision event observed when increasing K by one level. ARI and NMI quantify concordance between successive cuts and show that increasing K mainly refines pre-existing groups rather than inducing an abrupt reorganisation of pocket space. This table therefore supports the interpretation of K as a multi-resolution exploration parameter rather than as a uniquely optimal clustering threshold.

S2.2.2. Multi-criteria assessment of clustering resolution

To further assess clustering resolution in PockFlex, we performed a complementary multi-criteria analysis on the PR1 benchmark case using the same distance-based hierarchical clustering framework. Five complementary criteria were evaluated across K = 2–8: Silhouette, Dunn, resampling-based stability, Intra/Inter, and C-index. As shown in Figure S1, these criteria do not converge toward a single sharply dominant optimum, but rather support an informative intermediate regime around K = 4–5.

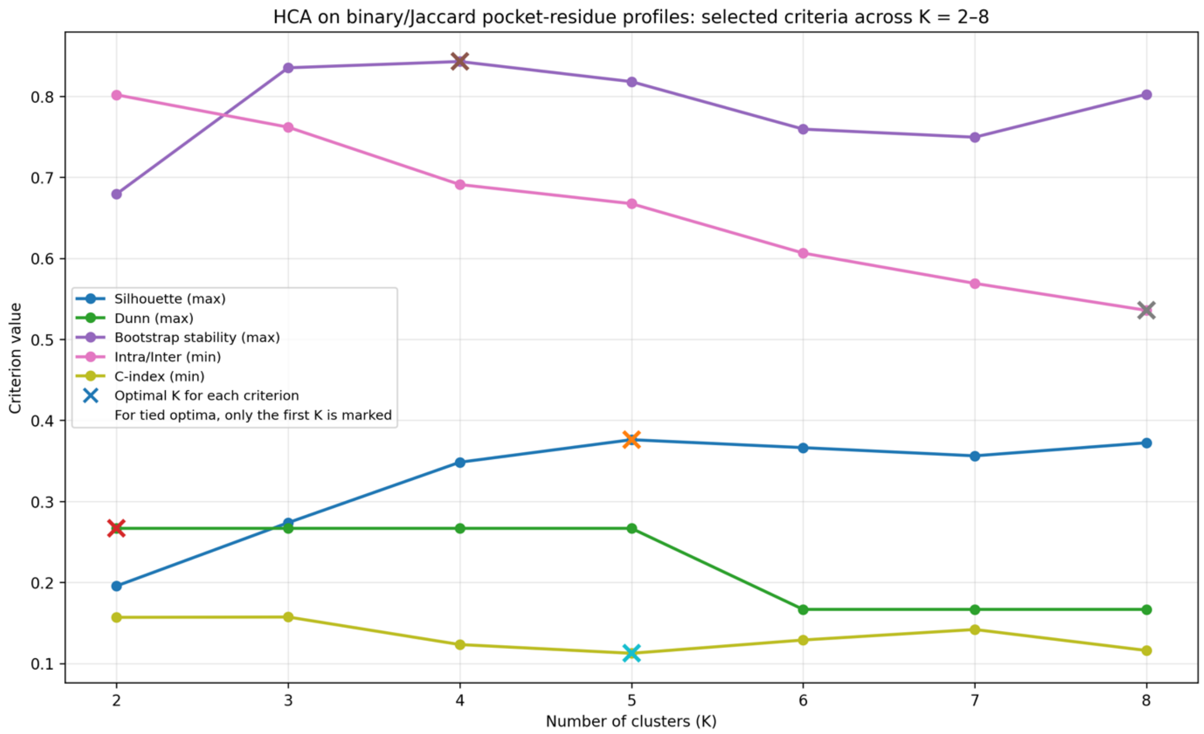

Crosses indicate the optimal K for each criterion; for tied optima, only the first K is marked.

Figure S1. Multi-criteria assessment of clustering resolution on the PR1 benchmark case. Five complementary criteria compatible with distance-based hierarchical clustering of binary residue profiles were evaluated across K = 2–8: Silhouette, Dunn, resampling-based stability, Intra/Inter, and C-index. Crosses indicate the optimal K for each criterion (maximum for Silhouette, Dunn, and resampling-based stability; minimum for Intra/Inter and C-index). Rather than identifying a single sharply dominant optimum, the figure supports an informative intermediate-resolution regime around K = 4–5, consistent with progressive hierarchical refinement of the same global pocket organisation.

This pattern is consistent with the hierarchical organisation observed in the reference dendrogram: lower K values provide a coarse-grained overview of the dominant pocket organisation, whereas higher K values mainly correspond to progressively finer subdivision of pre-existing clusters. Within this range, K = 4 was retained as the reference intermediate resolution for practical interpretation because it provides a simple and well-structured partition of the main pocket organisations while remaining easy to interpret. K = 5 would also have been a defensible choice, but corresponds to a slightly finer subdivision of the same global organisation. Concretely, this analysis does not support the idea of one uniquely “correct” number of clusters. Instead, it supports a stable intermediate-resolution regime in which K functions as an exploration parameter. Overall, these results support the interpretation of K as a resolution parameter within an exploratory multi-resolution workflow, rather than as a uniquely optimal estimate of the true number of binding-site clusters.

S2.2.3. Stability under conformational subsampling

To assess the robustness of the clustering workflow against structural noise and pocket-detection variability, we performed repeated conformational subsampling on the PR1 ensemble and reran the clustering workflow at K = 4 on each perturbed subset. Stability was assessed by subsampling conformations rather than residue features, because the concern here specifically relates to variability across closely related conformations. A total of 100 replicates were generated by randomly sampling 80% of the conformations without replacement. For each replicate, the resulting partition was compared with the reference partition restricted to the pockets present in that replicate. Global partition stability was evaluated using ARI and NMI, and the four major reference pocket clusters were tracked using best-match binary similarity; the resulting global and cluster-wise stability estimates are summarised in Table S3. Overall, the results showed high global stability of the clustering and robust recovery of the four major reference pocket clusters across subsampling replicates. Practically, this indicates that the main reconstructed PR1 binding-site clusters are not highly dependent on a specific subset of conformations. Even after moderate perturbation of the structural ensemble, the major clusters remain detectable and largely consistent with the reference partition. Taken together, these observations indicate that the main reconstructed binding-site clusters are unlikely to be driven primarily by pocket-detection variability and remain detectable under moderate perturbations of the structural ensemble.

Panel A. Global partition stability

Metric	Value
Subsampling replicates	100
Conformations retained per replicate	80%
Reference partition	K = 4
Median ARI [IQR]	0.856 [0.802-0.871]
Median NMI [IQR]	0.875 [0.836-0.888]

Panel B. Cluster-wise stability of the K=4 major pocket clusters

Reference cluster size	Median best-match binary [IQR]
375	0.889
313	0.881
144	0.992
109	1.000

Table S3. Stability of PR1 clustering under repeated conformational subsampling. PR1 clustering stability was evaluated over 100 subsampling replicates, each built from 80% of the conformations. Panel A summarises global partition stability relative to the reference clustering at K = 4 using ARI and NMI. Panel B reports cluster-wise stability for the four major reference pocket clusters using median best-match binary similarity across replicates. IQR, interquartile range. The table shows that the main reconstructed clusters remain robustly detectable under

moderate perturbation of the structural ensemble, supporting the view that they are not primarily driven by pocket-detection variability.

S2.3. Clustering parameterisation and justification

Beyond the resolution-oriented analyses reported in Note S2.2, we further examined the clustering formulation itself on the PR1 benchmark case, focusing on the effects of distance and linkage choices, as well as on possible biases related to pocket size or residue count. The aim here is not to claim that the chosen formulation is the only valid one, but to determine whether the main recovered organisation remains stable and interpretable under related methodological choices.

S2.3.1. Sensitivity to distance and linkage choices

To clarify the methodological behavior of the residue-based clustering strategy, we examined its sensitivity on the PR1 benchmark case with respect to distance formulation and linkage choice. All comparisons were performed on the same pocket-by-residue binary matrix derived from the PR1 dataset. At $K = 4$, retained here as an informative intermediate resolution, binary + Ward.D2 and euclidean + Ward.D2 yielded substantially concordant partitions, whereas changing the linkage from Ward.D2 to average at fixed binary distance produced a lower concordance. These results indicate that the overall organisation of residue-defined pocket space is relatively robust to the tested distance formulation, whereas the fine partitioning of pocket clusters is more sensitive to the agglomeration rule. Ward.D2 was therefore retained as the default clustering rule because it provided a compact and hierarchically structured organisation of recurrent pocket clusters in PR1, consistent with the intended multi-resolution exploration of binding sites. The corresponding dendrogram comparison is shown in Figure S2, and concordance metrics are summarised in Table S4.

Concretely, this section shows that the major organisation recovered by PockFlex is not an artefact of one narrowly tuned distance definition. However, it also indicates that the precise boundaries of finer subclusters depend more strongly on the linkage rule.

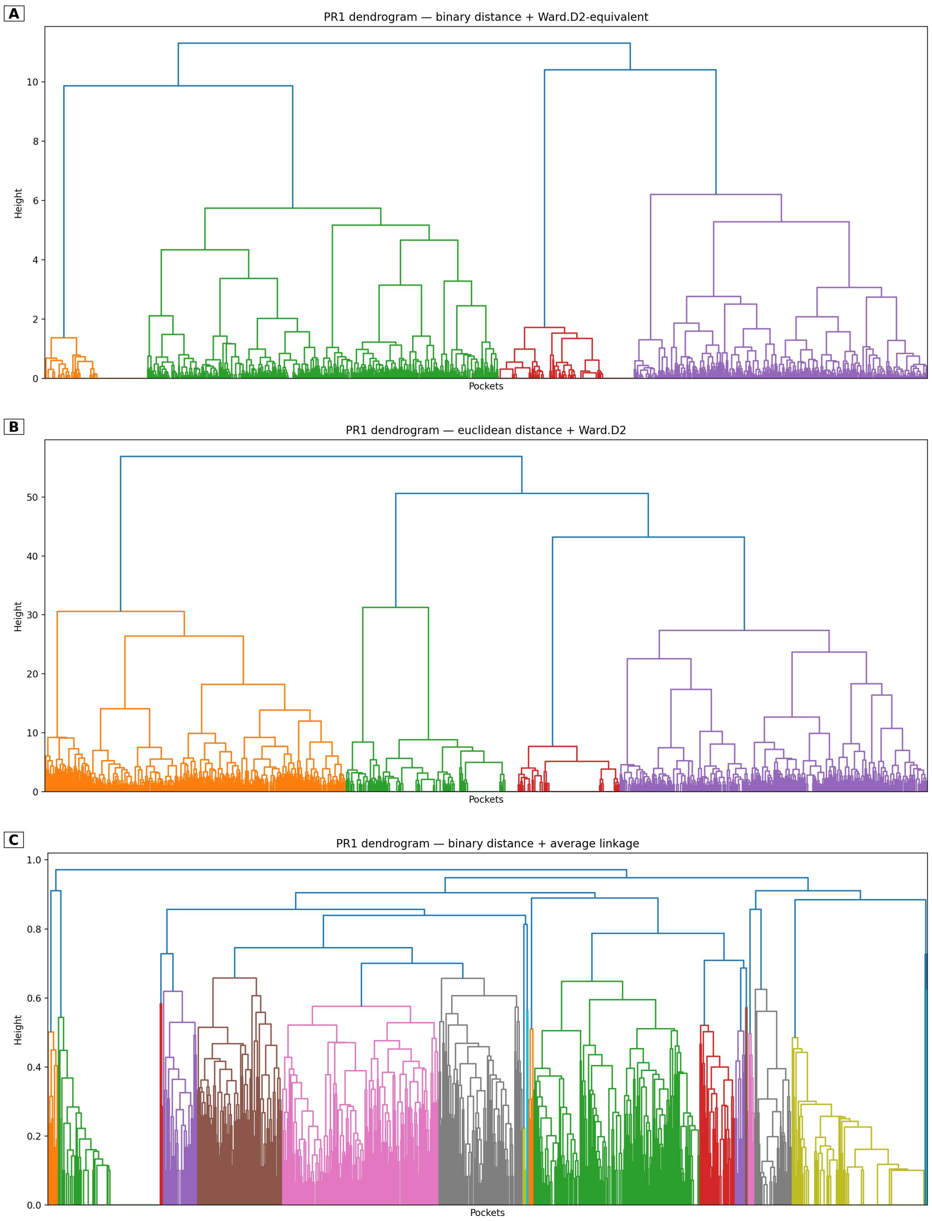

Figure S2. Multi-panel dendrogram comparison on PR1. Comparison of hierarchical clustering results obtained from the same PR1 pocket-by-residue matrix using three formulations: (A) binary + Ward.D2, (B) euclidean + Ward.D2, and (C) binary + average. The figure shows that the global residue-based organisation of pocket space is largely preserved across the two tested distance formulations, whereas the agglomeration rule has a stronger effect on the fine definition of cluster boundaries. This comparison supports the robustness of the main clustering structure while highlighting that high-resolution subcluster boundaries remain method-dependent.

Sensitivity analysis	Comparison	Result	Interpretation
Distance	binary + Ward.D2 vs euclidean + Ward.D2	ARI = 0.7467; NMI = 0.7856	Substantial concordance across tested distance formulations
Linkage	binary + Ward.D2 vs binary + average	ARI = 0.4575; NMI = 0.6466	Fine partition more sensitive to linkage choice

Table S4. Sensitivity of PR1 clustering to distance and linkage choices. Concordance between alternative clustering formulations was evaluated at K = 4 using the ARI and NMI. The comparison distinguishes the effect of the distance formulation from that of the agglomeration rule on the recovered clustering structure. The results indicate substantial concordance across tested distance formulations, whereas the finer partitioning of pocket clusters is more sensitive to linkage choice.

S2.3.2. Control for pocket-size and residue-count effects

To address the possibility that clustering might be biased by pocket size or residue count, we performed three complementary controls on PR1. First, we quantified the association between pairwise binary dissimilarities and absolute differences in residue count. Second, we compared the reference clustering with a partition based on residue count alone. Third, we restricted the analysis to pockets with similar residue counts and examined whether pockets assigned to the same global clusters remained closer to each other than pockets assigned to different clusters. Across these three controls, the results provided little evidence for a dominant size effect. The association between binary distance and residue-count differences was weak, the reference clustering showed very low concordance with a clustering based on residue count alone, and the separation between major pocket clusters remained detectable even within a size-restricted subset. The corresponding control results are summarised in Table S5. Practically, these controls show that the recovered PR1 clustering cannot be reduced to a simple ordering of pockets by size. Residue composition contributes substantially to the observed organisation, including when comparisons are restricted to pockets of similar size. Taken together, these analyses indicate that the recovered clustering is not reducible to pocket size or residue count alone and that residue composition contributes substantially to the observed clustering structure. Although this clustering formulation is not presented as the only possible choice and could be further explored in future work, these results support its use as a coherent and interpretable framework for grouping compositionally close pockets within the exploratory multi-resolution logic of PockFlex.

Analysis	Metric	Result	Interpretation
Size-effect control 1	Spearman correlation between binary distance and |Δcount|	ρ = -0.125	Weak association between distance structure and residue-count differences
Size-effect control 2	Reference clustering vs residue-count-only clustering	ARI = -0.022; NMI = 0.147	Clustering not reducible to pocket size alone
Size-effect control 3	Size-restricted subset (10–14 residues): mean intra vs inter distance	0.684 vs 0.960	Global clusters remain separated even when size variation is restricted

Table S5. Controls for pocket-size and residue-count effects on PR1. Three complementary controls were used to determine whether the reference clustering could be reduced to a simple effect of pocket size or residue count. These analyses include the association between binary distance and residue-count differences, comparison with a residue-count-only clustering, and intra- versus inter-cluster distances in a size-restricted subset. Taken together, the results provide little evidence for a dominant size effect and support the conclusion that residue composition, rather than residue count alone, substantially contributes to the recovered clustering structure.

S2.4. Integrated interpretation of clustering robustness

Taken together, these analyses support a robust multi-resolution interpretation of PockFlex clustering on PR1. The default K = 2 partition should be understood as a coarse-grained exploratory view rather than as a uniquely optimal clustering threshold. Increasing K mainly results in progressive hierarchical subdivision of the same major pocket clusters, rather than in an abrupt reorganisation of pocket space. The complementary criteria do not support a single uniquely dominant optimum, but rather an informative intermediate-resolution regime around K = 4–5. Within this range, K = 4 was retained as the main reference resolution for practical interpretation, whereas K = 5 represents a closely related but slightly finer partition, and K = 6 can be used to illustrate further hierarchical refinement. The residue-based organisation remains globally robust to the tested distance formulation, whereas fine cluster boundaries are more sensitive to the linkage criterion. Additional controls indicate that the recovered clustering is not reducible to pocket size or residue count alone. Finally, repeated conformational subsampling shows that the major reconstructed binding sites remain stable under moderate perturbations of the structural ensemble. Overall, the main methodological message is that the PR1 pocket clusters recovered by PockFlex behave as recurrent structural organisations that can be explored at several resolutions, rather than as unstable artefacts produced by a specific clustering choice. This supports the use of the workflow for structured ensemble-level interpretation of recurrent binding-site patterns, while still acknowledging that fine-grained boundaries may depend on the chosen level of resolution.

Supporting material S3. Practical use and interpretation of the PockFlex workflow

S3.1. Overview of the workflow

PockFlex is an ensemble-level, residue-centred framework designed to reconstruct recurrent binding-site clusters across structural ensembles. It does not require a predefined binding site as input. Instead, each conformation is first processed independently with the PockDrug workflow, in which candidate pockets are estimated using an Fpocket-based geometric procedure and assigned pocket-level druggability scores. Only pockets involving at least eight pocket-lining residues are retained for downstream analysis. The pockets overlapping the selected region of interest (ROI) are then grouped into recurrent clusters according to residue-level similarity across the ensemble. In this framework, the ROI should be understood as an exploration scope rather than as a predefined pocket. The main parameters and operational thresholds are summarised in Table S6.

S3.2. Defining the ROI as an exploration scope

The ROI corresponds to a user-defined set of residues delimiting the structural region used to retain and compare detected pockets after per-conformation pocket estimation. It should not be interpreted as a predefined pocket or binding site. Depending on the biological question, the ROI may be broad, for instance encompassing an entire protein, a domain, or a large interface region, or more focused, for instance targeting a candidate functional region. Thus, the ROI controls the scope of the analysis while leaving the actual pocket organisation to be reconstructed from the ensemble.

In practice, a broad ROI is useful when no specific site or subregion is assumed beforehand and when the goal is to obtain an initial overview of the accessible pocket landscape within a biologically or structurally relevant region. Conversely, a more focused ROI is appropriate when prior structural or functional information already points to a particular region of interest, such as an interface, a localized functional area, or a region selected after an initial exploratory run for closer examination. In both cases, the ROI should be understood as a way to delimit the structural search space rather than as evidence that a single binding site is already known.

S3.3. Interpreting and prioritising the initial clustering

When no specific site or subregion is assumed beforehand, a broad ROI provides an appropriate starting point. Pockets detected within this ROI are clustered according to residue-level similarity across the ensemble, and each resulting cluster should be interpreted as a recurrent binding-site pattern observed across multiple conformations. The default *low-K* setting is automatically suggested based on the average number of ROI-overlapping pockets detected per conformation. This value is intended only as an initial guide for multi-resolution exploration, while the final choice of K remains user-defined. Increasing K yields a finer partition, whereas decreasing K produces broader groupings.

Cluster interpretation should combine three complementary types of information: recurrence, residue-level coherence, and druggability. Recurrence reflects how often a given cluster is observed across the ensemble. Residue-frequency statistics help distinguish stable contributors from more flexible or peripheral ones. The druggability score is computed at pocket level within

the PockDrug workflow and summarised at cluster level as the proportion of pockets predicted to be druggable within the cluster. Clusters showing substantial recurrence together with a high prevalence of favourable conformations represent good candidates for downstream prioritisation, whereas less recurrent or less druggable clusters should be interpreted more cautiously. Thus, the first clustering should be viewed primarily as an exploratory overview of recurrent pocket organisations within the selected structural region. Within a given session, users may re-run the analysis with alternative K values to examine whether specific clusters remain stable or split into more localised subclusters at higher resolution.

Depending on the objective of the analysis, users may either inspect the full cluster partition or focus on a subset of clusters by applying frequency- and/or druggability-based filters, using thresholds defined in the [0,1] interval. Conversely, less recurrent or less druggable clusters may still remain informative when exploring alternative conformational states or transient binding opportunities. Users may also adjust the residue-frequency threshold used for display to highlight either highly recurrent residues or less frequent contributors within clusters. Because pockets are detected at the atomic level whereas clustering is performed at the residue level, pockets that differ slightly in atomic definition may still be grouped together when they share a similar residue composition. Overall, PockFlex helps users move from a collection of independently detected pockets to a structured, residue-level interpretation of recurrent binding-site organisation across structural ensembles.

S3.4. Iterative refinement of promising regions

PockFlex is designed for iterative analysis. A practical strategy is to begin with a broad ROI, identify recurrent and interpretable clusters, and then perform a second run on a more focused ROI centered on one promising region. This local refinement reduces interference between neighboring regions and provides a more resolved description of cluster variability within the selected area. Within the same session, users may successively explore several refined ROIs derived from the initial overview, depending on the regions they wish to examine in greater detail.

The residue-frequency threshold used for display can be adjusted to highlight either highly recurrent residues or less frequent contributors within clusters. Because pockets are initially detected at the atomic level whereas clustering is performed at the residue level, pockets that differ slightly in atomic definition may still be grouped into the same cluster when they share a similar residue composition. In this sense, PockFlex does not aim to redefine pocket geometry, but to stabilise and refine the interpretation of recurrent binding-site organisation across conformations.

S3.5. Key parameters and operational thresholds

The main required, user-defined, fixed, automatically initialised, and derived parameters used for pocket detection, clustering, and result interpretation are summarised in Table S6. These include the definition of the ROI, the maximum number of conformations considered in one run, the minimum pocket size retained for downstream clustering, the initialisation of the clustering parameter K, the minimum number of pockets per cluster retained in the standard summary, the residue display frequency threshold, and both pocket-level and cluster-level druggability descriptors.

Parameter	Definition / default use	Value / rule	Status
**Input structures**	Single compressed archive containing PDB or CIF structures with consistent residue numbering across the ensemble.	Required input condition	Required
**Region of interest (ROI)**	User-defined residue set delimiting the structural region used to retain and compare detected pockets after per-conformation pocket estimation; it may correspond to an entire protein, a domain, an interface region, or a focused functional region; the ROI is not a predefined pocket.	User-defined 1 to 300 residues	User-defined
**Maximum number of conformations**	Maximum number of conformations considered in one run; depends on ROI size to keep the analysis tractable.	30 to 500, depending on ROI length	User-defined
**Minimum pocket size retained**	Only pockets involving at least eight pocket-lining residues are retained for downstream ROI filtering and clustering.	≥ 8 residues	Fixed preprocessing filter
**Initial number of clusters (K)**	After pocket detection across the ensemble, an initial *K* value is heuristically suggested to guide clustering exploration; the final *K* remains user-defined.	Heuristic value: rounded average number of ROI-overlapping pockets detected per conformation	Heuristically suggested, chosen by the user
**Minimum number of pockets per cluster**	Minimum cluster size retained in the standard summary.	30	Fixed
**Residue display frequency threshold**	Threshold used to display stable or rarer residues within clusters in the outputs; can be tuned to emphasize recurrent residues or less frequent contributors.	75% by default	Automatically initialized, then user-adjustable
**Pocket-level druggability score**	Continuous PockDrug-based score available for each individual pocket.	*Score* d in [0,1]	Computed upstream
**Binary pocket druggability**	Operational interpretation of pocket-level druggability.	*Score* d ≥ 0.50: druggable	Fixed operational threshold
**Cluster-level druggability**	Summary descriptor reflecting the prevalence of favorable conformations within a cluster.	Proportion of cluster pockets with d ≥ 0.50	Fixed
**Druggability categories**	Qualitative interpretation of pocket-level druggability scores in the interface. Four predefined categories based on score d.	very druggable: d ≥ 0.75<br>quite druggable: 0.50 ≤ d < 0.75<br>less druggable: 0.25 ≤ d < 0.50<br>not druggable: d < 0.25	Fixed

Table S6. Key parameters and operational thresholds used in the PockFlex workflow. This table summarises the main required, user-defined, fixed, automatically initialised, and derived parameters used for pocket detection, clustering, and result interpretation in PockFlex. It is intended as a practical guide for first-pass exploration and iterative refinement of recurrent binding-site clusters across structural ensembles.

S3.6. Illustrative applications

The practical logic described above can be illustrated using previously published applications methodologically related to the workflow later formalised in PockFlex. These examples are provided as use cases to clarify how broad or focused ROIs, recurrent cluster interpretation, and iterative refinement can be combined in practice. They should be understood as illustrative applications rather than as a broad external benchmark.

S3.6.1. Influenza A virus NS1 RNA-binding domain

In the NS1 case study, the groove region of the RNA-binding domain provides an example of a focused ROI centered on a biologically defined interface region. Within this region, flexibility-aware pocket analysis and residue-based clustering support the distinction between a central recurrent groove site and more lateral, locally deformable pocket configurations. In practical terms, this example illustrates how a targeted ROI can be used to reconstruct recurrent pocket organisations within a known functional region while still distinguishing a dominant recurrent site from more local subsite heterogeneity.

S3.6.2. SARS-CoV-2 spike receptor-binding domain

In the SARS-CoV-2 receptor-binding domain case study, the analysis illustrates a broader exploratory strategy. A larger ROI covering the RBD surface can yield several recurrent pocket clusters with different localisations, frequencies, and druggability profiles. Subsequent interpretation then helps distinguish sites with distinct functional relevance, including

interprotomer regions, a fatty-acid-associated pocket, and a mutation-sensitive interface-related site. In practical terms, this example illustrates how an initial broad ROI can provide a global overview of recurrent pocket organisations, which can then be narrowed toward a smaller set of prioritised regions for more focused interpretation.

Overall, these illustrative applications highlight the intended use of PockFlex as a workflow that moves from ROI-based structural exploration to residue-level reconstruction, prioritisation, and refinement of recurrent binding-site patterns across conformational ensembles.

S3.6.3. PR1 benchmark case: HIV-1 protease from whole-protein exploration to focused ROI refinement

The PR1 benchmark case illustrates the iterative use of PockFlex in a concrete system. A first run with a whole-protein ROI provides a global overview of recurrent pocket organisation across the ensemble, whereas a second run focused on the central region enables a more resolved interpretation of local pocket states and variability. In this sense, PR1 exemplifies the intended progression from broad structural exploration to region-specific refinement. The corresponding application is presented in the main text, and a more detailed PR1 analysis is provided in the dedicated supporting material.

Supporting material S4. Detailed HIV-1 protease (PR1) pocket analysis

S4.1. PR1 system preparation and molecular dynamics simulation

HIV-1 protease (PR1) is a key enzyme in the viral life cycle and a major target for antiretroviral therapy. Its catalytic activity depends on large-amplitude flap motions that regulate access to the active site, and this conformational flexibility complicates inhibitor design. Characterising the diversity of pocket conformations explored by PR1 is therefore important for identifying alternative binding regions and for interpreting binding-site plasticity under dynamics.

S4.1.1. PR1 structure preparation

The starting structure was the unbound PR1 (PDB code: 1HHP), which contains one monomer. To reconstruct the biologically relevant dimer, we followed the procedure described by Triki et al. 2018 [4]. The symmetric dimer was generated in PyMOL using symmetry mates, and the resulting assembly was organised into chains A and B. To preserve an unambiguous distinction between the two monomers throughout the analysis, residues were renumbered sequentially, with chain A spanning residues 1–99 and chain B spanning residues 101–199.

Protonation states were assigned with PROPKA (version 3) [5]. All residues were kept in their standard protonation states expected at physiological pH, except Asp25 of chain B (Asp125 in the renumbered system), which was maintained in a deprotonated state to favor sampling of more open conformations.

S4.1.2. Molecular dynamics simulation

The MD simulation followed a protocol adapted from Badel et al.,2022 [6] with modifications regarding the starting structure and protonation state. Simulations were carried out with GROMACS (version 2024) [7] using the Amber ff99SB-ILDN force field [8]. The system was solvated in a dodecahedral TIP3P [9] water box with a minimum protein–box distance of 10 Å, and sodium and chloride ions were added to neutralise the system. After steepest-descent energy minimisation, the system was equilibrated through several restrained steps with progressively reduced positional restraints. Production MD was then run for 500 ns without positional restraints. Covalent bonds involving hydrogens were constrained with LINCS [10]. Long-range electrostatics were treated with Particle Mesh Ewald [11] using a 10 Å cutoff, and van der Waals interactions were computed with the same cutoff. Temperature was maintained at 310 K with the v-rescale thermostat, and pressure with the Parrinello–Rahman barostat. Frames were saved every 1 ns, yielding 501 conformations. To remove overall rotation and translation, frames were aligned on a reference structure extracted from the trajectory.

S4.2. Multi-resolution analysis of PR1 pockets with PockFlex

PockFlex was applied to the 501-frame PR1 ensemble to characterise recurrent pocket organisations and their variability across the trajectory to investigate the pocket landscape of the PR1, a well-established drug target whose catalytic activity depends on large-scale flap motions that modulate access to the active site [12-15].

S4.2.1. Broad ROI analysis

Consistent with the exploratory workflow of PockFlex, the first analysis was performed using a broad ROI encompassing the entire protein. In total, 2695 pockets were identified and clustered according to residue-composition similarity (Figure S3). This global classification revealed a structured and heterogeneous pocket landscape. One major cluster clearly corresponded to the central binding site, identified by the presence of catalytic residues. Importantly, this site was recovered without prior restriction to a local binding region. Several additional clusters also contained catalytic residues but differed in their surrounding residue composition, consistent with alternative conformational states of the central pocket associated with flap dynamics. In parallel, other clusters lacked catalytic residues and instead involved conserved regions such as the fulcrum and cantilever, highlighting alternative pocket organisations independent of the active site.

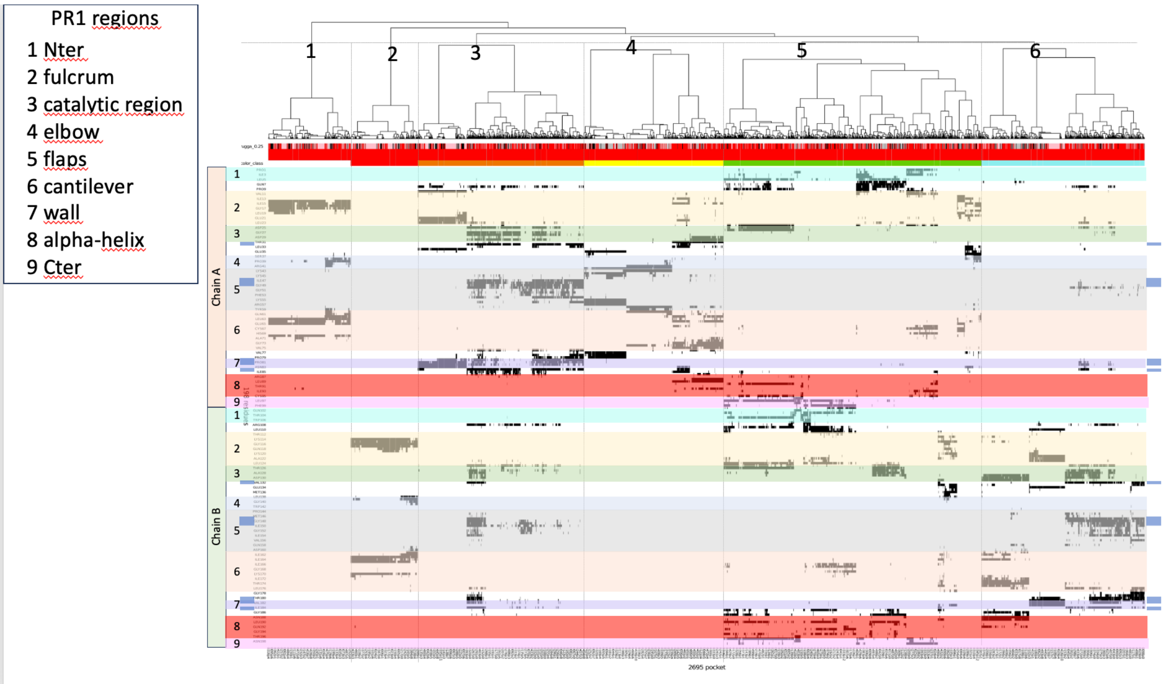

Figure S3. Hierarchical clustering of the 2695 pockets identified along the PR1 trajectory based on residue composition. Each column corresponds to a pocket, and each row represents a residue position for chains A and B. Black marks indicate the presence of a residue within a given pocket. The dendrogram at the top shows the hierarchical organisation of pockets, with six major clusters highlighted. The top bar reports pocket druggability scores, and the second bar indicates the six cluster assignments. Colored horizontal bands with transparency indicate the main structural regions of PR1 (N-terminal, fulcrum, catalytic region, elbow, flaps, cantilever, wall, α-helix, and C-terminal) as defined in Sadiq et al. [16], allowing visualisation of the contribution of each region to pocket composition. The blue rectangles displayed on the left and right sides highlight the residues included in the region of interest (ROI), used for the focused analysis of the central binding site.

S4.2.2. Focused ROI analysis of the central binding region

To examine the central binding site region in greater detail, a second analysis was performed using a focused ROI including 16 residues 32, 47–50, 80, 81, 84, 132, 147–150, 180, 181, and 184. A total of 1045 pockets were identified as ROI-overlapping, covering 91% of the frames of the trajectory. A first inspection of the dendrogram reveals the emergence of two main groups (clusters I and II), corresponding to pockets predominantly composed of residues from one or the other of the two chains, Figure 2. This separation reflects differences in residue composition between the two groups, with some pockets predominantly involving residues from one chain and others from the opposite chain.

Cutting the hierarchical classification at four clusters reveals that each of the two main groups further subdivides into one highly populated and one less populated subcluster. The two less populated subclusters (clusters 1 and 3) correspond to binding sites located in the fulcrum and wall regions. These pockets are oriented toward the exterior of the protein rather than toward the active site and are observed similarly in both chains A and B, reflecting the pseudo-symmetry of the dimer. They are present in 23% and 31% of the frames, respectively indicating that these pockets are regularly sampled throughout the trajectory and do not correspond to rare conformational events. In contrast to the central binding pocket, these clusters display a remarkably conserved residue composition, indicating limited structural variability. Their surface localisation and outward orientation suggest that they are unlikely to directly participate in substrate recognition. However, their proximity to the fulcrum, a region involved in the global dynamics of the protease, suggests that they may represent potential allosteric sites capable of modulating the conformational behavior of the protein. Thus, ROI-based selection does not exclusively isolate the central pocket, but also captures structurally distinct cavities that may correspond to potential allosteric sites.

S4.2.3. Six-cluster partition and conformational states of the central pocket

Refining the classification into six clusters resolved the conformational diversity of the central binding site. Two clusters corresponded to central pockets at the dimer interface. One represented a canonical symmetric state involving residues from both chains and observed in approximately 36% of frames. A second, more populated cluster corresponded to an asymmetric state dominated by one chain and observed in approximately 57% of frames. The symmetric state displayed a more conserved residue composition, whereas the asymmetric state showed greater variability, highlighting dynamic symmetry breaking within the homodimer [17]. These two clusters should not be interpreted as distinct binding sites, but rather as two major conformational states of the same central pocket: a relatively symmetric state involving both monomers and an asymmetric state dominated by one chain. This interpretation is consistent with asymmetric flap dynamics and indicates that the two monomers do not contribute equally to the central cavity throughout the trajectory.

S4.2.4. Recurrent lateral and chain-specific pockets

The remaining clusters corresponded to lateral or chain-specific pockets. One recurrent lateral pocket, observed predominantly in chain B and located at the flap–wall interface, showed a

highly conserved residue composition and was observed in approximately 37% of frames. Its location and recurrence are consistent with the previously described eye pocket, a lateral cavity proposed as a transient and potentially allosteric site in PR1 [18, 19].
The least populated cluster, observed in approximately 8% of frames, corresponded to a pocket located in the fulcrum and cantilever regions and restricted to a single chain. Despite its lower occurrence, its conserved residue composition indicates that it represents a well-defined structural state rather than random noise. Its limited population and specific localisation are consistent with a transient or cryptic cavity accessible only in a subset of conformations.

S4.2.5. Summary interpretation

Overall, the PR1 ensemble explored a diverse pocket landscape including: (i) a flexible central binding site adopting symmetric and asymmetric states linked to flap dynamics, (ii) recurrent lateral pockets with possible allosteric relevance, and (iii) low-populated chain-specific cavities compatible with transient or cryptic binding sites. These results highlight both the structural plasticity of the central site and a pronounced dynamic asymmetry between the two monomers.

S4.3. Comparison with D3Pockets

Chen et al. (2019) applied D3Pockets [20] to HIV-1 protease to characterise pocket stability, continuity, and geometric evolution along MD trajectories. Their analysis identified the central active site as the most prominent and functionally relevant pocket, while also detecting additional surface pockets with lower stability. They further showed that flap motions strongly modulate the geometry of the active site and can induce transient pocket reshaping or splitting. Our results are consistent with this general picture. In particular, we also recover the central binding site as a stable region whose geometry is strongly modulated by flap dynamics. However, the residue-based clustering strategy of PockFlex provides a complementary, state-resolved view of this behavior. Rather than describing only continuity or persistence, it distinguishes recurrent conformational states of the central pocket, including a relatively symmetric state and a more populated asymmetric state. In addition, it highlights recurrent lateral pockets and less populated chain-specific cavities that remain structurally well-defined when present. Thus, while D3Pockets emphasises geometric continuity and persistence, PockFlex provides a complementary interpretation centered on recurrent residue-defined pocket states across the ensemble. Together, these analyses support a view of HIV-1 protease as a highly dynamic system in which the central pocket and several alternative cavities are shaped by flap-dependent conformational variability.

References

1. Schmidtke, P., Bidon-Chanal, A., Luque, F. J., and Barril, X. MDpocket: open-source cavity detection and characterization on molecular dynamics trajectories. Bioinformatics, 27(23):3276–3285, 2011. https://doi.org/10.1093/bioinformatics/btr550
2. Kokh, D. B., Richter, S., Henrich, S., Czodrowski, P., Rippmann, F., and Wade, R. C. TRAPP: a tool for analysis of transient binding pockets in proteins. J. Chem. Inf. Model., 53(5):1235–1252, 2013. https://doi.org/10.1021/ci4000294
3. Chen, Z., Zhang, X., Peng, C., Wang, J., Xu, Z., Chen, K., Shi, J., and Zhu, W. D3Pockets: A Method and Web Server for Systematic Analysis of Protein Pocket Dynamics. J. Chem. Inf. Model., 59(8):3353–3358, 2019. https://doi.org/10.1021/acs.jcim.9b00332
4. Triki, D., Billot, T., Visseaux, B., Descamps, D., Flatters, D., Camproux, A.-C., and Regad, L. Exploration of the Effect of Sequence Variations Located inside the Binding Pocket of HIV-1 and HIV-2. Scientific Reports, 8:5789, 2018. https://doi.org/10.1038/s41598-018-24124-5
5. Olsson, M. H. M., Søndergaard, C. R., Rostkowski, M., and Jensen, J. H. PROPKA3: Consistent Treatment of Internal and Surface Residues in Empirical pKa Predictions. J. Chem. Theory Comput., 7(2):525–537, 2011. https://doi.org/10.1021/ct100578z
6. Badel, A., Breuil, L., Laville, P., and Regad, L. Exploration of the Structural Asymmetry Induced by the Intrinsic Flexibility of HIV-2 Protease. Symmetry, 14(2):362, 2022. https://doi.org/10.3390/sym14020362
7. Abraham, M. J., Murtola, T., Schulz, R., Páll, S., Smith, J. C., Hess, B., and Lindahl, E. GROMACS: High Performance Molecular Simulations through Multi-Level Parallelism from Laptops to Supercomputers. SoftwareX, 1–2:19–25, 2015. https://doi.org/10.1016/j.softx.2015.06.001
8. Lindorff-Larsen, K., Piana, S., Palmo, K., Maragakis, P., Klepeis, J. L., Dror, R. O., and Shaw, D. E. Improved Side-chain Torsion Potentials for the Amber ff99SB Protein Force Field. Proteins, 78(8):1950–1958, 2010. https://doi.org/10.1002/prot.22711
9. Jorgensen, W. L., Chandrasekhar, J., Madura, J. D., Impey, R. W., and Klein, M. L. Comparison of Simple Potential Functions for Simulating Liquid Water. J. Chem. Phys., 79(2):926–935, 1983. https://doi.org/10.1063/1.445869
10. Hess, B., Bekker, H., Berendsen, H. J. C., and Fraaije, J. G. E. M. LINCS: A Linear Constraint Solver for Molecular Simulations. J. Comput. Chem., 18(12):1463–1472, 1997. https://doi.org/10.1002/(SICI)1096-987X(199709)18:12<1463::AID-JCC4>3.0.CO;2-H
11. Essmann, U., Perera, L., Berkowitz, M. L., Darden, T., Lee, H., and Pedersen, L. G. A Smooth Particle Mesh Ewald Method. J. Chem. Phys., 103(19):8577–8593, 1995. https://doi.org/10.1063/1.470117
12. Kohl, N. E., Emini, E. A., Schleif, W. A., Davis, L. J., Heimbach, J. C., Dixon, R. A., Scolnick, E. M., and Sigal, I. S. Active Human Immunodeficiency Virus Protease Is Required for Viral Infectivity. Proc. Natl. Acad. Sci. U.S.A., 85(13):4686–4690, 1988. https://doi.org/10.1073/pnas.85.13.4686
13. Wlodawer, A., and Erickson, J. W. Structure-Based Inhibitors of HIV-1 Protease. Annu. Rev. Biochem., 62(1):543–585, 1993. https://doi.org/10.1146/annurev.bi.62.070193.002551
14. De Clercq, E. Antiretroviral Drugs. Curr. Opin. Pharmacol., 10(5):507–515, 2010. https://doi.org/10.1016/j.coph.2010.04.011
15. Menéndez-Arias, L., and Álvarez, M. Antiretroviral Therapy and Drug Resistance in Human Immunodeficiency Virus Type 2 Infection. Antiviral Research, 102:70–86, 2014. https://doi.org/10.1016/j.antiviral.2013.12.001
16. Sadiq, S. K., and De Fabritiis, G. Explicit Solvent Dynamics and Energetics of HIV-1 Protease Flap Opening and Closing. Proteins, 78(14):2873–2885, 2010. https://doi.org/10.1002/prot.22806
17. Triki, D., Cano Contreras, M. E., Flatters, D., Visseaux, B., Descamps, D., Camproux, A.-C., and Regad, L. Analysis of the HIV-2 Protease's Adaptation to Various Ligands: Characterization of Backbone Asymmetry Using a Structural Alphabet. Scientific Reports, 8:710, 2018. https://doi.org/10.1038/s41598-017-18941-3
18. Chen, Z., Zhang, X., Peng, C., Wang, J., Xu, Z., Chen, K., Shi, J., and Zhu, W. D3Pockets: A Method and Web Server for Systematic Analysis of Protein Pocket Dynamics. J. Chem. Inf. Model., 59(8):3353–3358, 2019. https://doi.org/10.1021/acs.jcim.9b00332

19. Damm, K. L., Ung, P. M. U., Quintero, J. J., Gestwicki, J. E., and Carlson, H. A. A Poke in the Eye: Inhibiting HIV-1 Protease through Its Flap-recognition Pocket. Biopolymers, 89(8):643–652, 2008. https://doi.org/10.1002/bip.20993
20. Ung, P. M.-U., Dunbar, J. B., Gestwicki, J. E., and Carlson, H. A. An Allosteric Modulator of HIV-1 Protease Shows Equipotent Inhibition of Wild-Type and Drug-Resistant Proteases. J. Med. Chem., 57(15):6468–6478, 2014. https://doi.org/10.1021/jm5008352
